# Efficacy of *Streptococcus salivarius* Blis K12 in the Prevention of Upper Respiratory Tract Infections in Physically Active Individuals: A Randomized Controlled Trial

**DOI:** 10.3390/microorganisms12112164

**Published:** 2024-10-26

**Authors:** Alexander Bertuccioli, Marco Cardinali, Matteo Micucci, Marco Bruno Luigi Rocchi, Chiara Maria Palazzi, Giordano Bruno Zonzini, Giosuè Annibalini, Annalisa Belli, Davide Sisti

**Affiliations:** 1 Department of Biomolecular Sciences, University of Urbino Carlo Bo, 61029 Urbino, Italy; alexander.bertuccioli@uniurb.it (A.B.); marco.cardinali@uniurb.it (M.C.); matteo.micucci@uniurb.it (M.M.); marco.rocchi@uniurb.it (M.B.L.R.); giordano.zonzini@uniurb.it (G.B.Z.); giosue.annibalini@uniurb.it (G.A.); a.belli7@campus.uniurb.it (A.B.); davide.sisti@uniurb.it (D.S.); 2 Microbiota International Clinical Society, 10123 Torino, Italy; 3 Department of Internal Medicine, Infermi Hospital, AUSL Romagna, 47921 Rimini, Italy

**Keywords:** probiotics, *Streptococcus salivarius*, upper respiratory tract infections

## Abstract

This study investigates the efficacy of *Streptococcus salivarius* K12 in preventing upper respiratory tract infections (URTIs) in healthy adults. URTIs are a common issue, particularly in physically active individuals, leading to significant disruptions in daily life. Probiotics, such as *S. salivarius* K12, have emerged as a potential preventive strategy for these infections. This research was conducted as a randomized, double-blind, placebo-controlled trial involving 112 participants aged between 19 and 25. Participants were randomly divided into two groups: one group received a daily dose of *S. salivarius* K12, marketed as Bactoblis^®^, while the other received a placebo. The trial lasted for four months, during which adherence to the treatment protocol was closely monitored. The primary goal was to measure the incidence of URTIs using the Jackson Scale and the Wisconsin Upper Respiratory Symptom Survey (WURSS-11). The results indicated that higher adherence to the *S. salivarius* K12 treatment was associated with an increased number of days without URTI symptoms. Although the overall severity of symptoms did not differ significantly between the treatment and control groups, those with high adherence to *S. salivarius* K12 (greater than 90%) reported more days free from illness. In conclusion, *S. salivarius* K12 demonstrated potential as a preventive measure against URTIs, especially in individuals who adhered strictly to the treatment regimen. However, further research involving larger populations and longer follow-up periods is needed to fully confirm these findings and better understand the role of *S. salivarius* K12 in preventing respiratory infections.

## 1. Introduction

Nutraceuticals, a term derived from “nutrition” and “pharmaceutical”, refers to food-derived products that provide health benefits beyond basic nutrition. The concept, first introduced in the late 20th century, encompasses a wide range of products, including dietary supplements and functional foods. These products, which occupy a clinical space bridging the gap between conventional foods and pharmaceuticals, often contain bioactive substances derived from food matrices. Such substances include phytocomplexes, phytochemicals, fibers, fatty acids, and probiotics, all of which may offer physiological benefits or reduce the risk of chronic diseases. As our understanding of the complex interactions between diet, food components, probiotics, health, and disease has evolved, there is a pressing need to elucidate the clinical efficacy and identify the determinants that may affect the clinical outcomes of these nutraceuticals.

The use of probiotics has evolved over time, transitioning from a general application for digestive health to more targeted and specific uses [1], thus defining the concept of “bacterial therapy” or “probiotic therapy” [2]. This shift has led to numerous applications beyond the digestive system, such as the prevention of strep throat infections, tonsillitis [2], oral caries [3], and upper respiratory tract infections (URTIs) [4]. *Streptococcus salivarius* K12 [5] is a key example of this evolution. This bacterium, isolated from the mouth of a healthy child, has been shown to counteract ß-hemolytic strains of *Streptococcus pyogenes* (Lancefield group A) [6,7] and other pathogenic bacteria like *Micrococcus luteus*, *Streptococcus anginosus*, *Eubacterium saburreum*, *Micromonas micros*, *Streptococcus pneumoniae*, *Haemophilus influenzae*, and *Moraxella catarrhalis* [8,9,10]. These effects are due to the ability to produce the cationic antimicrobial peptides (lantibiotics) Salivaricin A2 and Salivaricin B, encoded by the 190 kb megaplasmid present in the K12 strain [11]. These peptides are crucial for the bacterium’s ability to persistently colonize various tissues of the upper respiratory tract, as demonstrated in infants using the oral probiotic *S. salivarius* K12 [12,13]. This is important because intense physical stress, common in athletes, can increase susceptibility to infections, as described by the “open window” theory [14,15]. Scientific literature indicates a reduction in various immune system elements under these conditions [14,15], creating a favorable environment for the onset of URTIs [16]. Athletes under intense training stress [17,18] face a two- to six-times higher risk of URTIs following exposure to pathogens or environmental factors [19,20]. While *S. salivarius* K12 has shown promise in preventing [4] and treating URTIs and other respiratory infections, including SARS-CoV-2 [21], conflicting data exist. A study in New Zealand found a non-significant reduction in strep throat cases among children using K12, with more pronounced effects in older age groups [22]. Di Pierro noted several issues with the study, including suboptimal administration methods and diagnostic challenges [23]. Based on these conflicting data, our study aims to investigate the efficacy of *S. salivarius* K12 supplementation in adult subjects, examining whether and to what extent adherence to the treatment may influence its effectiveness. Overall, as reported in the literature, the use of probiotics like *S. salivarius* K12 plays an important preventive role in health, reducing the burden of infections and generally improving the quality of life.

## 2. Materials and Methods

### 2.1. Study Design

**Figure 1:**
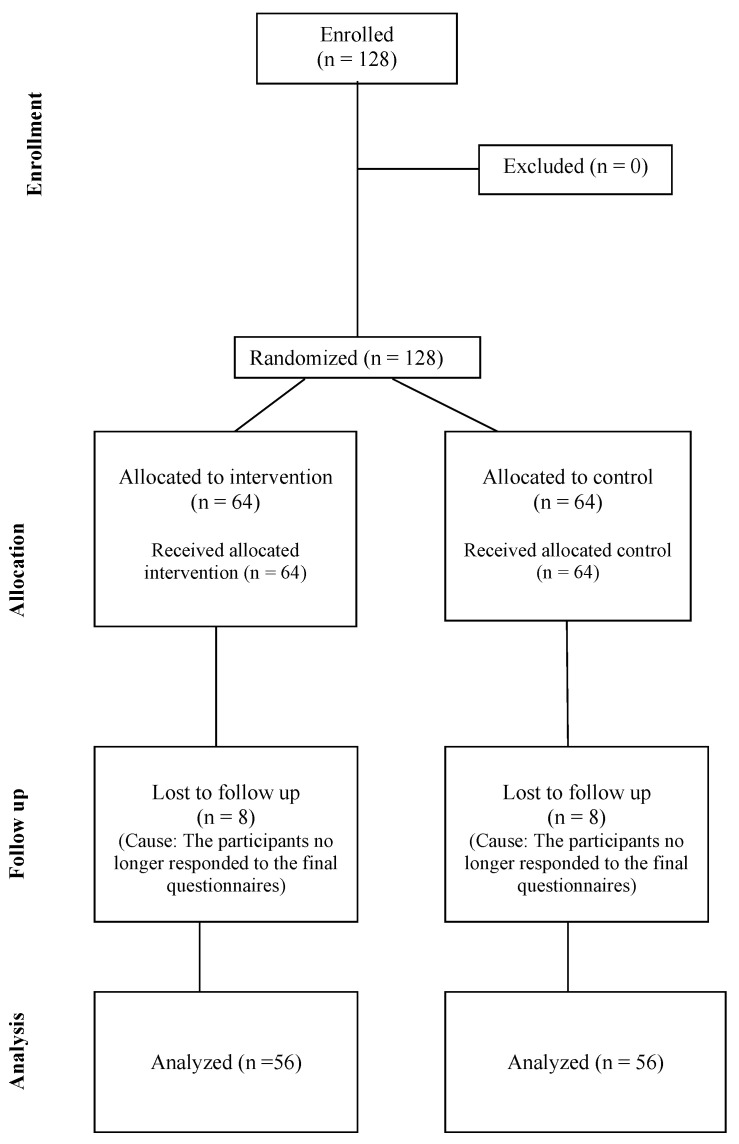
Flow diagram of the study.

This study was a double-blind controlled placebo study (RCT) involving 112 subjects (64 males and 48 females), aged between 19 and 25 years, for a four-month period with the primary aim of examining the effect of the supplementation with *Streptococcus salivarius* K12 (marketed in Italy as Bactoblis^®^—Pharmextracta SPA, Pontenure, PC, Italy, registration number in the national registry of food supplements 53435) on the incidence of URTIs. Secondary outcomes included the determination of type, duration and severity of URTIs episodes. The study was conducted by recruiting subjects from various track and field sports centers in the province of Pesaro and Urbino (PU), in the Marche region. Participants were randomly divided into two groups (treated and control) with randomization by permutation blocks (*n* = 4), stratified for gender. Subjects were instructed to take one tablet (Bactoblis^®^ or placebo) every night before bedtime, refraining from introducing food or liquids to facilitate the adherence and colonization of the strain in the oral mucosa. The placebo was a tablet identical in composition to Bactoblis^®^, except for the absence of microorganisms: it was indistinguishable in size, color, and taste. Each subject received instructions on product storage (temperature between +4 °C and +6 °C) (Figure 1).

### 2.2. Randomization

The study ratio was 1:1. The treatment was randomly assigned using a computer-generated random sequence. The order of treatment for each patient was communicated to all centers by the Urbino statistical unit. The order of assumption of Bactoblis/placebo was obtained by permuted-block randomization (size of block = 4) and patient stratification according to gender. The participants and raters were all blinded to treatment conditions.

### 2.3. Participants

Participants in the study were required to meet the following inclusion criteria: they had to be in good health at the commencement of the study, aged between 18 and 24 years. Additionally, participants must not have consumed any medications or probiotics in the four weeks before the study. They also agreed to abstain from consuming fermented milk products and other types of probiotics during the study period. The exclusion criteria for the study were as follows: smokers, individuals using medications for chronic conditions, and those consuming probiotics or fermented milk products, to eliminate potential confounding factors and ensure clean data. Participants with recurrent respiratory tract pathologies, pregnancy or breastfeeding, and previously diagnosed allergies or intolerances were also excluded. This study was conducted according to the principles stated in the Declaration of Helsinki and was approved by the Ethics Committee for Human Experimentation of Urbino University Carlo Bo (no. of approval 29_2020).

### 2.4. Product Characteristic

The product under evaluation (Bactoblis^®^) was registered as a dietary supplement in compliance with Italian law no. 169/2004. Notification to the Minister of Health was completed in July 2011 (registration no. 53435). Each tablet contained 5 billion CFU of *S. salivarius* K12 ATCC BAA-1024 (BLIS Technologies Ltd., Dunedin, New Zealand) at the time of production and was manufactured by S.I.I.T. (Trezzano sul Naviglio, Milan, Italy). The tablets were round, vanilla-flavored, and designed to dissolve slowly. The placebo was produced to match the active product in form, color, consistency, dissolution time, and flavor but did not contain probiotic bacteria.

### 2.5. Instruments

During this period, the presence of URTI symptoms was assessed daily using the Jackson Scale. Data collection was performed daily through a Google Forms link that participants were invited to complete; each evening, a reminder was sent to prompt participants to fill out the form. The Jackson Scale comprised 8 items: sneezing, nasal obstruction, nasal discharge, sore throat, cough, headache, chills, and malaise. Each item was rated on a 4-point Likert scale: 0 = absent, 1 = mild, 2 = moderate, 3 = severe. Only the first 4 items were specific to cold symptoms. The onset of an illness episode was defined by a “yes” response to the question “Do you think you are getting a cold?” for two consecutive days; the episode was considered over when the subject responded “no” to the question “Do you think you are still sick?” for two consecutive days. In the event of URTIs’ presence, the Wisconsin Upper Respiratory Symptom Survey (WURSS-11) was completed daily to assess symptom severity until the illness resolved. Daily symptoms were categorized as “healthy”, “very slightly”, “slightly”, “moderately”, and “severely” [24]. The sum of 9 items was considered as the total score. Participants were encouraged to express themselves honestly and to contact the study coordinators in case of any issues.

### 2.6. Statistical Analyses

The number of subjects to be recruited was set at 45 subjects per group and this choice was based on an expected rate of 2.0 ± 1.0 URTI episodes (M ± SD) during the winter months [25], a target 30% reduction in number of episodes, statistical power of 80%, and a Type I error of 5%, with a Cohen-d effect size of 0.6 (medium-effect). Finally, we aimed to recruit 128 volunteers to account for an estimated 30% dropout rate over the study period.

One hundred and twelve healthy young adults were recruited for the study and were randomly assigned in equal numbers to either the treatment or control group. Descriptive statistics were calculated for demographic and anthropometric characteristics, using an independent samples *t*-test. Adherence to the treatment protocol was closely monitored; the distribution of adherence values was visualized using a violin plot to illustrate the spread and central tendency of adherence in both the control and treatment groups. A two-way MANOVA (Multivariate Analysis of Variance) was conducted to examine the effects of treatment condition (treatment/control) and adherence level, with interaction, on the percentage of days categorized according to the WURSS-11 questionnaire. The response variable was transformed using the arcsine transformation. The arcsin_square root transformation was applied to normalize the distribution of proportion data. It also stabilized the variance across the range of proportions, meeting the MANOVA assumption of homoscedasticity. The effect sizes for post hoc comparison between groups were calculated according to Cohen [26], reporting Cohen’s d ES with 95% CI. Statistical analyses were performed using SPSS (version 22, IBM Corp., Armonk, NY, USA) or R studio software (version 2024.04.2)

## 3. Results

**Figure 2:**
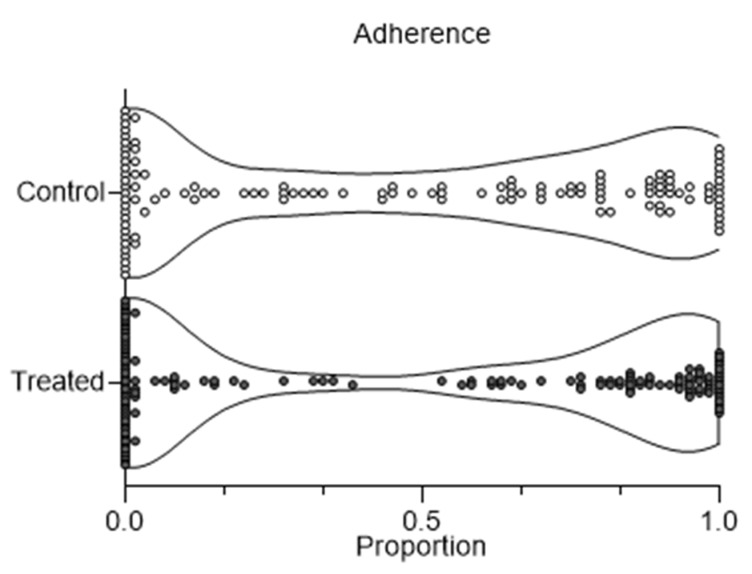
Violin plot of adherence of experimental groups (control and treated).

One hundred and twelve healthy young adults completed the experimental protocol, over a four-month period (from January to April) in 2023. The demographic and anthropometric characteristics of the entire participant group were as follows: age of 21.4 ± 2.4 years, height of 173.4 ± 10.7 cm, weight of 71.8 ± 13.2 kg, BMI of 23.6 ± 2.9 kg/m^2^, with females comprising 42.9% of the total sample. Table 1 shows the baseline characteristics of the two groups, considering that the treated group included 33 males and 23 females, while the control group included 31 males and 23 females. Due to the high adherence to treatment, the analysis of results was conducted considering factors obtained from the stratification of the adherence values. The sample distribution of adherence is reported above (Figure 2). No unintended effects were observed.

**Figure 3:**
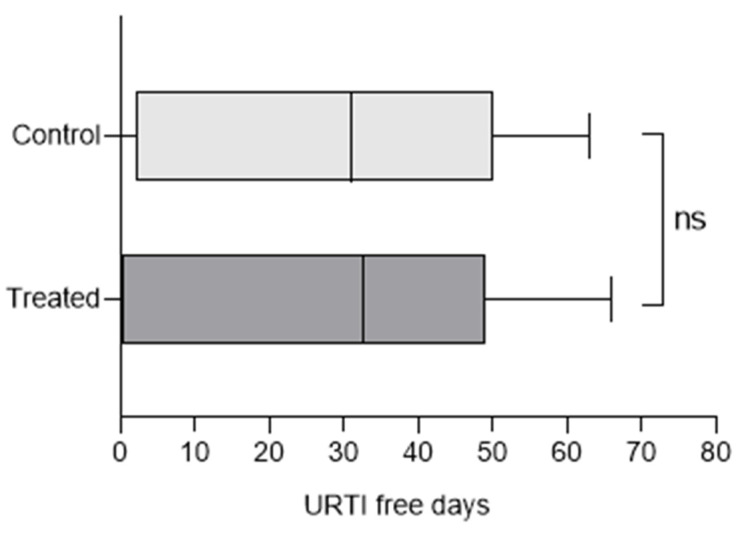
Box plot of URTI-free days of control and treated groups.

The enrolled subjects could be roughly divided into those who followed the daily intake dosage (>90%), those who followed it discontinuously (between 70 and 90%), and those who did not follow it (<70%). Participants were categorized into three adherence levels: <70%, 70–90%, and >90%. The adherence median values were 75 and 73%, respectively, from the control and treated group, showing a comparable median distribution; the first and third quartile were 10 and 95% for the control and 0 and 95% for the treated group. It is notable that the violin plot shows a strong bimodal shape distribution; indeed, enrolled subjects were either fully compliant or non-compliant. Considering the effect of treatment independently of the treatment adherence itself, it can be observed that the disease-free days in the upper respiratory tract were 75 and 73% of the days (see Figure 3); this proportion is not significant (*t*-test; *p* > 0.05).

**Figure 4:**
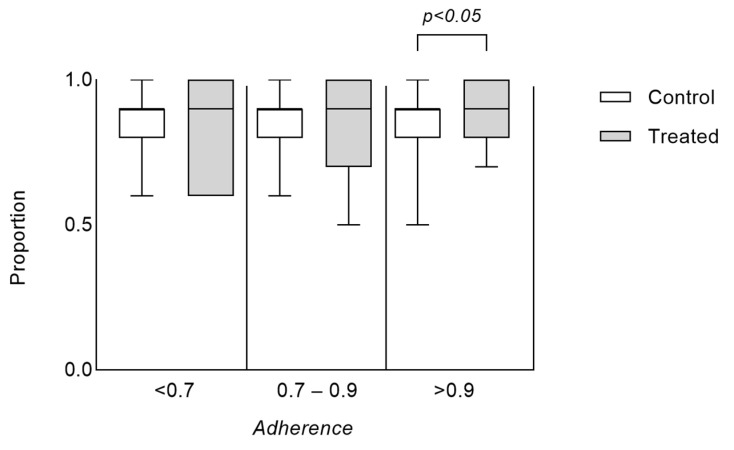
Statistics for the proportion of days free from illness across different adherence levels and treatment conditions.

The descriptive statistics for the proportion of days free from illness across different adherence levels and treatment conditions are summarized in Figure 4. For the < 70% adherence group, the mean proportion was 0.843 ± 0.127 in the control group and 0.828 ± 0.170 in the treatment group (*p* > 0.05; Cohen’s d ES = 0.09; 95%CI: −0.28–0.48). In the 70–90% adherence group, the mean was 0.859 ± 0.116 for the control and 0.827 ± 0.168 for the treatment group (*p* > 0.05; Cohen’s d ES = 0.22; 95%CI: −0.16–0.60). For the > 90% adherence group, the control group’s mean was 0.825 ± 0.115, while the treatment group’s mean was 0.892 ± 0.115 (*p* < 0.05; Cohen’s d ES = 0.58; 95%CI: 0.19–0.97) (Table 2). Median values were consistently high (≥0.90) across all groups, indicating a generally high proportion of illness-free days. The ranges (Min–Max) varied slightly, with the greatest range observed in the 70–90% treatment group (0.50) and the smallest in the >90% treatment group (0.30).

Descriptive statistics showed that the mean arcsine-transformed percentage of days free from illness varied slightly across adherence levels, with a higher adherence associated with slightly higher mean values in the treatment group compared to the control group.

A MANOVA was conducted to examine the differences in various dependent variables (Healthy, Very Slightly, Slightly, Moderately, and Severely) based on the independent variables of adherence and Control_treated, including their interaction.

Healthy: The analysis showed that the variable ‘Healthy’ was significantly influenced by adherence, with F(2, 264) = 321.403, *p* < 0.001, and a partial eta squared (η^2^) of 0.709, indicating a large effect size. The interaction effect between adherence and Control_treated was also significant, F(2, 264) = 5.224, *p* = 0.006, η^2^ = 0.038, suggesting that the effect of adherence on ‘Healthy’ varies depending on the treatment condition.

Very Slightly: For the variable ‘Very Slightly’, adherence had a significant effect, F(2, 264) = 23.899, *p* < 0.001, η^2^ = 0.153, reflecting a moderate effect size. However, the interaction between adherence and Control_treated was not significant, F(2, 264) = 2.205, *p* = 0.112, η^2^ = 0.016, indicating that the effect of adherence was consistent across different treatment conditions.

Slightly: The variable ‘Slightly’ was significantly affected by adherence, F(2, 264) = 26.066, *p* < 0.001, η^2^ = 0.165, which is a moderate effect size. There was no significant interaction between adherence and Control_treated, F(2, 264) = 1.594, *p* = 0.205, η^2^ = 0.012, suggesting that the differences due to adherence were not dependent on the treatment.

Moderately: The analysis revealed that adherence significantly influenced the ‘Moderately’ variable, F(2, 264) = 10.259, *p* < 0.001, η^2^ = 0.072, indicating a small to moderate effect size. The interaction between adherence and Control_treated was not significant, F(2, 264) = 1.899, *p* = 0.152, η^2^ = 0.014, implying that adherence’s effect was independent of the treatment condition. In the same way, for the variable ‘Severely’, neither adherence, F(2, 264) = 2.305, *p* = 0.102, η^2^ = 0.017, nor the interaction between adherence and Control_treated, F(2, 264) = 1.314, *p* = 0.270, η^2^ = 0.010, were significant.

Interaction Effects: The interaction between adherence and Control_treated was significant only for the ‘Healthy’ variable (η^2^ = 0.038), suggesting that the impact of adherence on health status varied depending on the treatment condition.

These results provide insights into the severity of the disease in both control and treated groups, segmented by their adherence to treatment across different percentages. Regardless of the treated and control groups, as well as the adherence percentage, there were no significant differences in the severity of symptoms related to URTIs; this is likely due to the low incidence of symptoms and the large standard deviations.

The Jackson Scale was utilized to evaluate cold symptoms; analysis of the data revealed no significant differences in the Jackson Scale scores between the control and intervention groups (*p* > 0.05). Additionally, when stratified by adherence to supplementation, no significant differences were observed within the subgroups (*p* > 0.05). These findings suggest that neither the group assignment nor the adherence category influenced the severity of cold symptoms as measured by the Jackson Scale.

The MANOVA results indicate significant effects of adherence on most health-related variables, with notable interaction effects for the ‘Healthy’ category. These findings highlight the importance of considering both adherence and treatment conditions when evaluating health outcomes.

## 4. Discussion

Over the years, the precision approach to probiotic therapy has been explored for various applications [27,28]. It is essential to explore the potential of *Streptococcus salivarius* K12 in infection prevention, as recent studies highlight its effectiveness against various infections. A key determinant of success in this context is therapeutic adherence; in fact, consistent adherence with a high profile is a determinant of the effectiveness of protection due to the fact that, as previously demonstrated, colonization is a transient phenomenon [12].

Hence, the primary aim of this study has been to comprehend the pivotal role that adherence plays in unlocking its protective effects against infectious diseases. In a study by Manning et al. [29], *Streptococcus salivarius* K12 demonstrates significant efficacy against bacterial infections, notably *Streptococcus pneumoniae*. This study reveals that pneumococcal growth could be prevented through megaplasmid-encoded bacteriocins and notably bacterial adherence to pharyngeal epithelial cells results inhibited by the presence of *S. Salivarius* K12. Chen et al. [30] elucidate the molecular mechanisms of *S. salivarius* BLIS K12 interaction with *S. pneumoniae*, highlighting strategies such as adhesion interference and disruption of vital processes. Various bacteriocin-specific mechanisms contribute to a reduced susceptibility to *Moraxella catarrhalis* infection. Laws et al. [31] investigate the immunomodulatory effects of *S. salivarius* BLIS K12, including downregulation of the NF-KB pathway and induction of beneficial pathways leading to type I and type II interferon release. This modulation affects the release of cytokines such as IL-6, IL-8, IL-10, IL-12, and TNF-α, and increases regulatory T cell (Treg) frequency, suggesting complex immune regulation. Consistent with these observations, the findings of Di Pierro et al. [21] highlighted a significant reduction in mortality among COVID-19 patients following the administration of *S. salivarius* BLIS K12 as an adjunct to standard care. Tagg’s analysis [5] emphasizes *S. salivarius* and its bacteriocin-like inhibitory substances (BLISs), showcasing distinctive antibacterial characteristics in prototype strains. According to these studies, Manti et al. [32], showed that bacteriotherapy with *S. salivarius* 24SMB and *S. oralis* 89a nasal spray represents an effective treatment for URTIs in children. This treatment reduces symptoms of URTIs, such as fever, cough, bronchospasm, rhinorrhea, and otalgia. Additionally, biofilm formation inhibition may help reduce the incidence and/or severity of acute otitis media and secretory otitis media [33]. Several studies report the effectiveness of the oral probiotic *S. salivarius K12* on streptococcal and viral pharyngotonsillitis [34] in children, as well as on the onset and severity of secretory otitis media (SOM) [35]. The research highlights that *S. salivarius K12* has shown promising results in the treatment of halitosis [36] and it has been shown to inhibit the production of IL-6 and IL-8 by gingival fibroblasts when activated by periodontal pathogens [37]. Despite the possibility that various factors such as diet [38], physical activity [39] and overall lifestyle [40] could contribute to infectious disease incidence reduction, the development of precision probiotic therapies offers an additional tool to enhance patient response. Based on previous studies, there is evidence of a protective effect against infectious diseases, making it essential to explore the factors that distinguish responders from non-responders. According to our data, therapeutic adherence emerges as a crucial factor, with adherence correlating to a 90% increase in the likelihood of achieving a protective effect. This suggests the importance of closely monitoring adherence in patients receiving treatment with *S. salivarius* K12, as high adherence could further enhance the protective effects against infections, thereby improving overall clinical outcomes. In general, as seen and demonstrated, probiotic therapy shows preventive effects in managing infectious diseases; however, it is important to underline some limitations of our study, such as the relatively small sample size and the duration of the follow-up.

Nevertheless, probiotic therapy should not be equated with various types of pharmacological treatments or prophylactic strategies such as vaccines; rather, it should be viewed as a complementary approach that can enhance patients’ responses to pathogens.

## 5. Conclusions

The diagnosis, treatment choice, and duration of URTIs pose significant challenges for physicians and are a relevant socio-economic issue, as highlighted by the SARS-CoV-2 pandemic. While *S. salivarius* K12 has been proposed for the prevention and treatment of URTIs, previous studies have shown conflicting results. This work highlighted how a four-month bedtime administration of Bactoblis^®^ tablet (*S. salivarius* K12) could achieve a protective effect, resulting in the high adherence group having a higher mean percentage of days free from illnesses in comparison to the control group.

Larger studies are required to confirm these results and determine the actual magnitude of the effect of *S. salivarius* K12 administration, as well as to identify further factors distinguishing responders from non-responders. Nonetheless, this bacterium continues to prove itself as a valuable resource for physicians.

## Figures and Tables

**Table 1:** Baseline characteristics of the two groups, expressed as mean ± standard deviation (SD) with relatives t value and *p* value in the third column.

	Treated (*n* = 56)	Control (*n* = 56)	t (*p*)
Age (years)	21.2 ± 2.5	21.6 ± 2.3	0.948 (0.372)
Weight (Kg)	71.2 ± 13.2	72.4 ± 13.3	1.211 (0.231)
Height (cm)	174.0 ± 10.6	172.6 ± 10.8	1.347 (0.184)
BMI (Kg/m^2^)	23.7 ± 2.8	23.5 ± 3.0	0.020 (0.8414)

**Table 2:** Days (expressed as mean ± SD) reported for the variables “Very Slightly”, “Slightly”, “Moderately”, “Severely” based on the group (treated/control) and adherence to treatment.

Adherence	Group	Very Sightly	Sightly	Moderately	Severely
>90%	Control	4.13 (5.17)	1.85 (2.51)	1.03 (2.23)	0.3 (0.16)
	Treated	3.74 (3.96)	2.44 (2.82)	0.77 (1.55)	0.10 (0.50)
70–90%	Control	1.83 (2.53)	1.00 (1.73)	0.26 (0.78)	0.03 (0.17)
	Treated	3.23 (4.73)	1.34 (2.42)	0.77 (1.70)	0.00 (0.00)
<70%	Control	1.28 (2.91)	0.55 (1.23)	0.20 (0.69)	0.01 (0.12)
	Treated	0.79 (2.86)	0.20 (1.01)	0.13 (0.48)	0.00 (0.00)

## Data Availability

The original contributions presented in the study are included in the article, further inquiries can be directed to the corresponding author. The CONSORT checklist is available in the Supplementary Materials Files.

## Supplementary Materials

The following supporting information can be downloaded at: https://www.mdpi.com/article/10.3390/microorganisms12112164/s1.

## References

[1] Veiga P., Suez J., Derrien M., Elinav E. (2020). Moving from Probiotics to Precision Probiotics. Nat. Microbiol..

[2] Di Pierro F., Adami T., Rapacioli G., Giardini N., Streitberger C. (2013). Clinical Evaluation of the Oral Probiotic *Streptococcus salivarius* K12 in the Prevention of Recurrent Pharyngitis and/or Tonsillitis Caused by *Streptococcus pyogenes* in Adults. Expert Opin. Biol. Ther..

[3] Babina K., Salikhova D., Polyakova M., Svitich O., Samoylikov R., Ahmad El-Abed S., Zaytsev A., Novozhilova N. (2022). The Effect of Oral Probiotics (*Streptococcus salivarius* K12) on the Salivary Level of Secretory Immunoglobulin A, Salivation Rate, and Oral Biofilm: A Pilot Randomized Clinical Trial. Nutrients.

[4] Bertuccioli A., Gervasi M., Annibalini G., Binato B., Perroni F., Rocchi M.B.L., Sisti D., Amatori S. (2023). Use of *Streptococcus salivarius* K12 in Supporting the Mucosal Immune Function of Active Young Subjects: A Randomised Double-Blind Study. Front. Immunol..

[5] Tagg J. (2004). Prevention of Streptococcal Pharyngitis by Anti-*Streptococcus pyogenes* Bacteriocin-like Inhibitory Substances (BLIS) Produced by *Streptococcus salivarius*. Indian J. Med. Res..

[6] Jack R.W., Tagg J.R., Ray B. (1995). Bacteriocins of Gram-Positive Bacteria. Microbiol. Rev..

[7] Sharma S., Verma K.K. (2001). Skin and Soft Tissue Infection. Indian J. Pediatr..

[8] Burton J.P., Chilcott C.N., Moore C.J., Speiser G., Tagg J.R. (2006). A Preliminary Study of the Effect of Probiotic *Streptococcus salivarius* K12 on Oral Malodour Parameters. J. Appl. Microbiol..

[9] Burton J., Chilcott C., Tagg J. (2005). The Rationale and Potential for the Reduction of Oral Malodour Using *Streptococcus salivarius* Probiotics. Oral Dis..

[10] Di Pierro F., Colombo M., Giuliani M.G., Danza M.L., Basile I., Bollani T., Conti A.M., Zanvit A., Rottoli A.S. (2016). Effect of Administration of *Streptococcus salivarius* K12 on the Occurrence of Streptococcal Pharyngo-Tonsillitis, Scarlet Fever and Acute Otitis Media in 3 Years Old Children. Eur. Rev. Med. Pharmacol. Sci..

[11] Hyink O., Wescombe P.A., Upton M., Ragland N., Burton J.P., Tagg J.R. (2007). Salivaricin A2 and the Novel Lantibiotic Salivaricin B Are Encoded at Adjacent Loci on a 190-Kilobase Transmissible Megaplasmid in the Oral Probiotic Strain *Streptococcus salivarius* K12. Appl. Environ. Microbiol..

[12] Horz H.-P., Meinelt A., Houben B., Conrads G. (2007). Distribution and Persistence of Probiotic *Streptococcus salivarius* K12 in the Human Oral Cavity as Determined by Real-Time Quantitative Polymerase Chain Reaction. Oral Microbiol. Immunol..

[13] Power D.A., Burton J.P., Chilcott C.N., Dawes P.J., Tagg J.R. (2008). Preliminary Investigations of the Colonisation of Upper Respiratory Tract Tissues of Infants Using a Paediatric Formulation of the Oral Probiotic *Streptococcus salivarius* K12. Eur. J. Clin. Microbiol. Infect. Dis..

[14] Souza D., Vale A.F., Silva A., Araújo M.A.S., De Paula Júnior C.A., De Lira C.A.B., Ramirez-Campillo R., Martins W., Gentil P. (2021). Acute and Chronic Effects of Interval Training on the Immune System: A Systematic Review with Meta-Analysis. Biology.

[15] Campbell J.P., Turner J.E. (2018). Debunking the Myth of Exercise-Induced Immune Suppression: Redefining the Impact of Exercise on Immunological Health Across the Lifespan. Front. Immunol..

[16] Miko B.A., Pereira M.R., Safdar A., Safdar A. (2019). Respiratory Tract Infections: Sinusitis, Bronchitis, and Pneumonia. Principles and Practice of Transplant Infectious Diseases.

[17] Cicchella A., Stefanelli C., Massaro M. (2021). Upper Respiratory Tract Infections in Sport and the Immune System Response. A Review. Biology.

[18] Nieman D.C. (1997). Risk of Upper Respiratory Tract Infection in Athletes: An Epidemiologic and Immunologic Perspective. J. Athl. Train..

[19] Åkerström T.C.A., Pedersen B.K. (2007). Strategies to Enhance Immune Function for Marathon Runners: What Can Be Done?. Sports Med..

[20] Lin L., Decker C.F. (2010). Respiratory Tract Infections in Athletes. Dis. Mon..

[21] Di Pierro F., Iqtadar S., Mumtaz S.U., Bertuccioli A., Recchia M., Zerbinati N., Khan A. (2022). Clinical Effects of *Streptococcus salivarius* K12 in Hospitalized COVID-19 Patients: Results of a Preliminary Study. Microorganisms.

[22] Doyle H., Pierse N., Tiatia R., Williamson D., Baker M., Crane J. (2018). Effect of Oral Probiotic *Streptococcus salivarius* K12 on Group A Streptococcus Pharyngitis: A Pragmatic Trial in Schools. Pediatr. Infect. Dis. J..

[23] Di Pierro F. (2019). Assessment of Efficacy of BLIS-Producing Probiotic K12 for the Prevention of Group A Streptococcus Pharyngitis: A Short Communication. Probiotics Antimicrob Proteins.

[24] Barrett B., Brown R.L., Mundt M.P., Thomas G.R., Barlow S.K., Highstrom A.D., Bahrainian M. (2009). Validation of a Short Form Wisconsin Upper Respiratory Symptom Survey (WURSS-21). Health Qual. Life Outcomes.

[25] Gleeson M., Bishop N.C., Oliveira M., Tauler P. (2011). Daily Probiotic’s (*Lactobacillus casei* Shirota) Reduction of Infection Incidence in Athletes. Int. J. Sport Nutr. Exerc. Metab..

[26] Cohen J. (1992). A Power Primer. Psychol. Bull..

[27] Di Pierro F., Bertuccioli A., Saponara M., Ivaldi L. (2020). Impact of a Two-Bacterial-Strain Formula, Containing *Bifidobacterium animalis* Lactis BB-12 and *Enterococcus faecium* L3, Administered before and after Therapy for *Helicobacter pylori* Eradication. Minerva Gastroenterol. Dietol..

[28] Di Pierro F., Bertuccioli A., Pane M., Ivaldi L. (2020). Effects of Rifaximin-Resistant *Bifidobacterium longum* W11 in Subjects with Symptomatic Uncomplicated Diverticular Disease Treated with Rifaximin. Minerva Gastroenterol. Dietol..

[29] Manning J., Dunne E.M., Wescombe P.A., Hale J.D.F., Mulholland E.K., Tagg J.R., Robins-Browne R.M., Satzke C. (2016). Investigation of *Streptococcus salivarius*-Mediated Inhibition of Pneumococcal Adherence to Pharyngeal Epithelial Cells. BMC Microbiol..

[30] Chen T.Y., Hale J.D.F., Tagg J.R., Jain R., Voss A.L., Mills N., Best E.J., Stevenson D.S., Bird P.A., Walls T. (2021). In Vitro Inhibition of Clinical Isolates of Otitis Media Pathogens by the Probiotic *Streptococcus salivarius* BLIS K12. Probiotics Antimicrob. Proteins.

[31] Laws G.L., Hale J.D.F., Kemp R.A. (2021). Human Systemic Immune Response to Ingestion of the Oral Probiotic *Streptococcus salivarius* BLIS K12. Probiotics Antimicrob. Proteins.

[32] Manti S., Parisi G.F., Papale M., Licari A., Salpietro C., Miraglia Del Giudice M., Marseglia G.L., Leonardi S. (2020). Bacteriotherapy with *Streptococcus salivarius* 24SMB and *Streptococcus oralis* 89a Nasal Spray for Treatment of Upper Respiratory Tract Infections in Children: A Pilot Study on Short-Term Efficacy. Ital. J. Pediatr..

[33] Bidossi A., De Grandi R., Toscano M., Bottagisio M., De Vecchi E., Gelardi M., Drago L. (2018). Probiotics *Streptococcus salivarius* 24SMB and *Streptococcus oralis* 89a Interfere with Biofilm Formation of Pathogens of the Upper Respiratory Tract. BMC Infect. Dis..

[34] Di Pierro F., Colombo M., Zanvit A., Risso P., Rottoli A.S. (2014). Use of *Streptococcus salivarius* K12 in the Prevention of Streptococcal and Viral Pharyngotonsillitis in Children. Drug Healthc. Patient Saf..

[35] Zupancic K., Kriksic V., Kovacevic I., Kovacevic D. (2017). Influence of Oral Probiotic *Streptococcus salivarius* K12 on Ear and Oral Cavity Health in Humans: Systematic Review. Probiotics Antimicrob. Proteins.

[36] Yoo H.-J., Jwa S.-K., Kim D.-H., Ji Y.-J. (2020). Inhibitory Effect of *Streptococcus salivarius* K12 and M18 on Halitosis In Vitro. Clin. Exp. Dent. Res..

[37] MacDonald K.W., Chanyi R.M., Macklaim J.M., Cadieux P.A., Reid G., Burton J.P. (2021). *Streptococcus salivarius* Inhibits Immune Activation by Periodontal Disease Pathogens. BMC Oral Health.

[38] Bertuccioli A., Ninfali P. (2014). The Mediterranean Diet in the Era of Globalization: The Need to Support Knowledge of Healthy Dietary Factors in the New socio-Economical Framework. Mediterr. J. Nutr. Metab..

[39] Chastin S.F.M., Abaraogu U., Bourgois J.G., Dall P.M., Darnborough J., Duncan E., Dumortier J., Pavón D.J., McParland J., Roberts N.J. (2021). Effects of Regular Physical Activity on the Immune System, Vaccination and Risk of Community-Acquired Infectious Disease in the General Population: Systematic Review and Meta-Analysis. Sports Med..

[40] Saberi Hosnijeh F., Kolijn P.M., Casabonne D., Nieters A., Solans M., Naudin S., Ferrari P., Mckay J.D., Weiderpass E., Perduca V. (2020). Mediating Effect of Soluble B-Cell Activation Immune Markers on the Association between Anthropometric and Lifestyle Factors and Lymphoma Development. Sci. Rep..

